# The Comprehensive Assessment of Social Media Use: Development and Validation Study

**DOI:** 10.2196/87599

**Published:** 2026-04-29

**Authors:** Nathan Job Lowry, Rebecca R Gebert, Zachary Rogers, Christine B Cha, Colleen M Jacobson

**Affiliations:** 1 Department of Counseling and Clinical Psychology, Teachers College, Columbia University, 525 West 120th Street, New York, NY, 10027, United States, 1 212-678-3000; 2 Department of Psychological & Brain Sciences, Boston University, Boston, MA, United States; 3 Child Study Center, School of Medicine, Yale University, New Haven, CT, United States; 4 Continuum Psychiatric Services, LLP, New York, NY, United States

**Keywords:** social media, young adults, scale development, survey measurement, mental health

## Abstract

**Background:**

Nearly all youth use the internet daily, with many maintaining several social media accounts. As increasing numbers of young people engage online and the ways we communicate fundamentally change, it is increasingly important to ask: how do these shifts influence youth mental health? To better understand how social media may affect mental health, researchers require validated tools that capture young people’s heterogeneous experiences with social media. However, few available measures evaluate the full range of positive and negative behaviors associated with its use, limiting our ability to meaningfully advance interventions promoting online hygiene.

**Objective:**

This study aims to develop and validate the Comprehensive Assessment of Social Media Use (CASM). The CASM is a self-report survey measure that moves beyond simple duration or frequency of use and captures how young people engage with social media. Importantly, the CASM assesses both the positive and negative dimensions of social media engagement.

**Methods:**

Two studies are outlined in this paper. Study 1 outlines the process of item generation and exploratory factor analysis. Study 2 outlines confirmatory factor analysis and validity testing. Both studies were conducted online and enrolled a convenience sample of college-aged young adults. Study 1 enrolled 260 participants (mean age 19.73, SD 2.91; n=172, 66.2% female; n=164, 63.1% White; n=38, 14.6% lesbian, gay, bisexual, transgender/transsexual, and queer [LGBTQ]). Study 2 enrolled 508 participants (mean age 18.99, SD 1.17; n=323, 63.6% female; n=272, 53.5% White; n=58, 11.4% LGBTQ).

**Results:**

Exploratory and confirmatory factor analysis resulted in a 29-item CASM scale that assesses 7 distinct aspects of young adult social media use: self-branding, compulsive use, disruptive use, impulsive sharing, social engagement, induce negative emotions, and induce positive emotions. This model accounted for 61% of the variance in responses. The chi-squared test of model fit was significant (*χ*²_356_=941, *P*<.001; root mean square error of approximation=0.064; comparative fit index=0.855; Tucker-Lewis index=0.848; standardized root mean squared residual=0.060). Factor internal consistency reliability ranged from 0.699 to 0.817. Validity testing suggested moderate discriminant, convergent, and criterion validity.

**Conclusions:**

The CASM measures a broad range of social media behaviors, enabling researchers to more effectively examine associations between online engagement and mental health outcomes. We hope the CASM will help researchers better understand how young people interact with social media, and that this knowledge will inform the development of more targeted interventions promoting healthy online habits.

## Introduction

Social media is a ubiquitous part of life in the United States, particularly among teenagers and young adults [1]. Nearly all teenagers (96%) use the internet daily, with many reporting spending at least 4 hours on their phones [2]. Most of this time is spent on social media apps, such as YouTube, TikTok, Instagram, and Snapchat [1]. Importantly, this increased time spent online is reshaping youth friendships and social development by providing immediate connection with peers, easy access and sharing of information, and a distinct digital social environment with its own cues and customs [3,4]. However, young people’s screen use is not homogeneous. Variations across age, gender, race, and other social factors influence one’s motivations to visit these digital spaces and their subsequent experiences there [5,6]. As social media continues to redefine how we interact with one another, and as increasing numbers of young people struggle to disconnect despite recognizing their excessive use [1], it is increasingly important to ask: how do these changes influence youth mental well-being and how can we best measure young people’s heterogeneous experiences with social media?

A considerable amount of research has found associations between social media use and negative mental health outcomes [7,8]. Problematic social media use (ie, patterns of engagement that disrupt daily functioning) has been identified as a distinct and meaningful type of social media use associated with anxiety, attention problems, and increased risk of self-harm and suicidality [9]. Recently, surges in screen time and online social engagement during the COVID-19 pandemic [10,11] have been linked to amplified mental health risks associated with problematic social media use in the years that followed [9,12]. As such, more nuanced study of social media use patterns in the post–COVID-19 era (normative, problematic, healthy, or otherwise) is especially important.

To better understand how social media may impact mental well-being, researchers need validated measures that examine how an individual engages with these online platforms. Many studies have measured associations between social media use and mental health in terms of quantity (ie, the number of hours spent on social media), though correlations are generally weak or not significant [13-16]. Time spent online has received criticism as a stand-alone metric to measure social media use because a strictly quantitative approach does not consider the context or content of use [17]. More recent efforts examining qualitative differences in social media use (eg, posting vs browsing, seeking out frustrating content vs happy content) reveal stronger associations with indicators of mental well-being [5,18,19]. A popular dichotomy that has emerged from this research is that which differentiates passive social media use (eg, “lurking” or “scrolling”) from active use (eg, creating content or directly communicating with other users) [20]. These studies generally assert that passive use is more associated with negative mental health outcomes [21-23], though null associations [24], individual differences [25], and inconsistencies across contexts [20,26] cast doubt on whether a passive-active distinction alone can predict poor outcomes from social media use.

Several instruments have been developed to assess social media use behaviors. For example, the Social Media Addiction Questionnaire [27] and the Social Media Disorder Scale [28] focus on user motivations and addictive characteristics of social media such as loss of control, tolerance, withdrawal, and conflict. Measuring social media behaviors that mirror addiction characteristics can identify unique risk factors for problematic use [29,30]. However, addiction-centric measures omit other ways in which an individual may engage with a digital environment, namely those that are healthy and adaptive [5,31]. They also overlook subjective aspects of social media, such as one’s perceived social investment or emotional affect during use [3,4]. Alternatively, the Social Media Use Scale [32] analyzes functional dimensions of social media use (ie, sharing beliefs, making social comparisons, managing social image, and consuming content). The focus on broader categories of behavior is likely to more accurately capture how individuals engage with online social platforms; however, positive and prosocial dimensions of social media engagement are still absent from this measure. Further, this scale measures the frequency of specific behaviors, which may be susceptible to recall bias. To gain a more holistic understanding of social media use and its associations with mental well-being, researchers could benefit from a tool that simultaneously assesses a broad range of both positive and negative social media behaviors.

This study outlines the development and validation of the Comprehensive Assessment of Social Media Use (CASM), a survey measure that assesses several aspects of young adult social media use. Given the shortcomings of prior efforts to effectively measure social media behavior, the CASM is structured around self-appraisal, reporter agreement with items (rather than duration or frequency reports) and the integration of positive as well as negative qualities of social media use. Two studies were conducted, hereafter referred to as study 1 and study 2. Study 1 outlines the process of item generation and exploratory factor analysis. Study 2 outlines the process of confirmatory factor analysis and validity testing.

## Study 1

### Overview

The goal of study 1 was to first create a comprehensive list of candidate items to assess a range of social media behaviors. The authors first drafted items to assess both positive and negative aspects of social media use, including addictive characteristics (eg, *How hard is it to resist the urge to check social media?*), self-branding (eg, *How much do your profiles represent your “real life”?*), adaptive coping (eg, *How often do you turn to social media to relax?*), and positive uses (eg, *How often do you use social media for activism purposes?*). Additional candidate items were then created after reviewing previous research and measures examining social media behaviors [33-37] to ensure a representative list of survey questions. Psychology professors at a small liberal arts college from a variety of disciplines (eg, cognitive, school, and clinical) informally reviewed the items and provided feedback. Since many young people have multiple social media accounts [1,2], all items use flexible language that is not specific to one social platform’s culture or cues. To respond, participants indicate the extent to which they agree with an item’s statement using a Likert scale ranging from 1 (not often or not at all) to 10 (very often or very much), such that a higher score indicates greater perceived engagement in a specific social media behavior. Our item generation process created a total of 42 candidate items. We then aimed to conduct an exploratory factor analysis to identify latent variables that would guide the structure of the CASM.

### Methods

#### Participants

We enrolled a convenience sample of college-aged young adults. Participants were recruited from introductory psychology classes at a small liberal arts college in the Northeast United States. If applicable, individuals could be compensated for participation with research credit hours for a class requirement. Study flyers promoting the study were also shared on social media. To participate, individuals had to be between 18 and 29 years old, be fluent in English, and certify that they could provide informed consent.

Study 1 enrolled a sample of 260 participants (Table 1). Participants were predominantly female (n=172, 66.2%), White (n=164, 63.1%), and non-LGBTQ (lesbian, gay, bisexual, transgender/transsexual, and queer; n=215, 82.7%) with a mean age of 19.73 (SD 2.91) years. All participants owned at least 1 social media account (mean 4.9, SD 1.8) and reported spending an average of 5.3 (SD 4.1) hours per day on social media.

**Table 1.** Participant demographics.

Demographics	Study 1 (n=260)	Study 2 (n=508)
Age (y), mean (SD; range)	19.73 (2.9; 18‐29)	18.99 (1.2; 18‐24)
Gender, n (%)		
Male	85 (32.7)	182 (35.8)
Female	172 (66.2)	323 (63.6)
Identify as LGBTQ^a^, n (%)		
Yes	38 (14.6)	58 (11.4)
No	215 (82.7)	438 (86.2)
Race or ethnicity, n (%)		
White	164 (63.1)	272 (53.5)
Black	28 (10.8)	73 (14.4)
Hispanic Latino	48 (18.5)	129 (25.4)
Asian	4 (1.5)	12 (2.4)
Middle Eastern	2 (0.8)	6 (1.2)
Other or unknown	13 (5)	14 (2.8)
Number of social media accounts, mean (SD; range)	4.9 (1.8; 1‐15)	4.6 (1.5; 0‐11)
Hours per day on social media, mean (SD; range)	5.3 (4.1; 0‐21)	5.4 (3.7; 0‐21)

a LGBTQ: lesbian, gay, bisexual, transgender/transsexual, and queer.

#### Procedures

Data collection occurred from April 2018 to November 2018 on the online research platform Qualtrics. After providing informed consent, participants answered a brief demographics survey. Participants then completed the 42 CASM candidate items. After completing the study measures, participants received a debriefing form with a list of local and national mental health resources.

#### Data Analysis

Descriptive statistics were calculated to characterize sample demographics. An exploratory factor analysis was conducted to identify item groupings for survey subscales and determine which candidate items to retain. Principal axis factoring with oblique rotation was used to allow for correlations to exist between factors. To retain an item, the following criteria were used, as recommended by Costello and Osborne [38]: (1) items must have a factor loading greater than 0.40, (2) any items that cross-loaded with a value greater than 50% of the largest factor value were eliminated, and (3) any factors with less than 3 items were removed. For all analyses, missing data were handled using listwise deletion. All statistical analyses were conducted with SPSS (IBM).

#### Ethical Considerations

This study received institutional review board approval from Iona College (reference number: 201718‐22) in 2018. Before participating, individuals agreed to participation by electronically signing a consent form that outlined study aims, procedures, time requirements, potential risks, and rights for human research subjects. Individuals from special or protected groups (ie, individuals who are <18 years old, imprisoned, or cognitively impaired) with impaired ability to provide free consent were ineligible to participate. We did not screen for pregnancy, and pregnant women were considered eligible to participate.

### Results

The first exploratory factor analysis produced a solution that included 12 factors that accounted for 67.5% of the variance. Using the criteria outlined above, a total of 16 items were eliminated, resulting in a 6-factor, 26-item solution. We deviated from the established cutoff criteria on 1 occasion: factors 7 and 9 were retained as we felt they would combine into 1 factor since both contained items related to online social communication. Thus, 8 factors and 30 items were retained from the first exploratory factor analysis.

A second exploratory factor analysis was conducted to confirm the identified factors. Using the same criteria outlined by Costello and Osborne [38], the second exploratory factor analysis resulted in a 7-factor solution with 29 items that accounted for 61.1% of the variance. One minor adjustment was made to the solution: item 29 (*How often do you post to make others think you are feeling better than you actually are?*) was moved to factor 6, as it had previously loaded onto factor 6 and aligned more with its theme of inducing negative affect. The factors and their internal consistency reliability are as follows: self-branding (0.829), compulsive use (0.798), disruptive use (0.759), impulsive sharing (0.701), social engagement (0.755), induce negative emotions (0.768), and induce positive emotions (0.735). Table 2 lists all retained items and their factor loading, mean, and SD.

**Table 2.** Factor loadings, means, and SDs for the final, 7-factor scale from study 1 exploratory factor analysis (EFA)

Factor or item	Factor loading							Mean (SD)
	1^a^	2^b^	3^c^	4^d^	5^e^	6^f^	7^g^	
Self-branding								
How much do your profiles represent an “idealized self”?	0.51	0.06	0.17	0.03	0.06	0.07	0.23	4.78 (2.66)
How often do you put a filter on pictures that you post or share?	0.51	0.19	−0.08	−0.05	−0.01	0.07	0.07	5.41 (3.16)
How important to you is the number of likes you get on a post?	0.79	−0.04	−0.09	0.09	−0.02	−0.1	0.1	4.82 (2.8)
To what extent do you post on a schedule/certain time of day, thinking people will be more likely to see your post?	0.79	−0.1	0.04	−0.16	−0.02	0.07	0.15	4.73 (3.31)
To what extent do you have a process/routine to developing your posts before sharing them?	0.64	0	0.05	−0.01	0.08	0.14	−0.02	3.71 (2.75)
How much does the number of likes you get on a post affect your mood?	0.53	0.04	0.11	0.11	0.09	0.12	0.1	3.63 (2.58)
Compulsive use								
How often do you check social media during face-to-face conversations?	−0.02	0.42	0.11	0.23	−0.08	−0.16	0.04	3.26 (1.94)
How often do you spend more time on social media than you intended to?	0.1	0.86	0	0.24	0.01	0.19	0.1	5.55 (2.59)
How often do you sleep with your phone within arm’s reach?	0.05	0.48	−0.02	0.06	0.03	−0.07	0.01	8.53 (2.54)
How often do you stop doing other tasks (such as homework) to check social media?	0.07	0.7	0.07	−0.07	−0.07	−0.02	0.11	6.75 (2.59)
How often do you lose track of time because you get so involved in your social media activity?	−0.05	0.68	0.15	−0.17	0.08	0.08	0.08	5.32 (2.85)
Disruptive use								
How hard is it to not check social media during class?	0.11	0.15	0.46	0.08	−0.01	0.05	−0.17	4.14 (2.95)
How often has a loved one (parent, friends, etc.) told you that you are on social media too often?	−0.07	0.06	0.51	0.19	0.01	0.01	0.16	3.41 (2.54)
How often have you gotten “in trouble” in class or at work for using social media when you shouldn’t have been?	−0.16	−0.02	0.78	0.08	0.14	0.15	−0.04	2.26 (1.71)
How likely are you to use (check or post) social media accounts while driving?	0.05	0.1	0.42	0.24	0.08	0.03	0.24	2.11 (1.85)
How uncomfortable do you feel if you are away from your phone and can’t use social media for an extended period of time (i.e., one h or more)?	0.18	0.17	0.42	0.07	0	0.1	0.01	3.82 (2.57)
Impulsive sharing								
How often have you felt that you “over-shared” on social media?	0	−0.09	0.26	0.58	0.1	−0.08	0.01	2.6 (2.17)
How often have you posted something on a social media site and later regretted it?	0.21	−0.11	0.12	0.49	0.04	0.06	0.06	2.97 (2.13)
How often do you post on social media as a reaction to stress?	0.17	0.01	0.12	0.67	0.08	0.04	0.27	3.05 (2.76)
Social engagement								
How often do you use social media to stay connected for extracurriculars?	0.11	−0.1	0.11	−0.14	0.73	−0.13	0.05	4.56 (2.59)
How often do you use social media for information about school/academics?	0.02	0.09	0.24	0.11	0.63	0.04	0.21	4.86 (2.82)
How often do you use social media for activism purposes?	−0.1	0.15	−0.16	0.23	0.7	0.07	−0.07	3.61 (2.86)
How often do you engage in discourse about social issues/justice over social media?	−0.01	0.09	−0.13	0.34	0.53	0.02	−0.11	3.06 (2.55)
Induce negative emotions								
Do you ever spend time viewing profiles even though you know it makes you feel badly about yourself?	0.16	0.06	0.04	0.07	0	0.63	−0.08	4.35 (3.07)
How often do you post to make others think you are feeling better than you actually are?	0.03	−0.16	0.04	0.61	−0.07	0.29	0.17	3.83 (2.78)
How often do you look at social media pages that you know will make you feel bad/induce negative affect?	0.07	−0.04	0.09	0.1	−0.09	0.7	−0.03	2.91 (2.51)
Induce positive emotions								
How often do you turn to social media because you think it will improve your mood?	0.04	0.13	0.09	0.4	0.07	0.13	0.56	4.6 (2.95)
How often do you turn to social media to relax?	0.07	0.23	−0.13	0.17	0.03	−0.13	0.57	5.62 (2.79)
How much does your social media activity make you feel connected to others?	0.29	0.01	−0.02	0.05	0.14	−0.03	0.45	5.43 (2.55)

a Eigenvalue: 8.92; percentage of variance: 29.73; Cronbach α: 0.83.

b Eigenvalue: 2.19; percentage of variance: 7.31; Cronbach α: 0.8.

c Eigenvalue: 1.76; percentage of variance: 5.86; Cronbach α: 0.76.

d Eigenvalue: 1.68; percentage of variance: 5.61; Cronbach α: 0.7.

e Eigenvalue: 1.54; percentage of variance: 5.13; Cronbach α: 0.76.

f Eigenvalue: 1.14; percentage of variance: 3.81; Cronbach α: 0.77.

g Eigenvalue: 1.08; percentage of variance: 3.61; Cronbach α: 0.74.

## Study 2

### Overview

The goal of study 2 was to conduct a confirmatory factor analysis to evaluate the model fit of the 29-item CASM measure created in study 1 (see Multimedia Appendix 1). A secondary goal was to assess the discriminant, convergent, and criterion validity of the CASM.

### Methods

#### Participants

We enrolled a convenience sample of college-aged young adults. Sample recruitment and inclusion criteria are identical to those outlined in study 1. Study 2 enrolled 508 participants (Table 1). Participants were predominantly female (n=323, 63.6%), White (n=272, 53.5%), and non-LGBTQ (n=438, 86.2%) with a mean age of 18.99 (SD 1.17) years. All participants used at least 1 social media account (mean 4.55, SD 1.54), and individuals reported spending an average of 5.35 hours per day on social media (SD 3.72).

#### Measures

##### Comprehensive Assessment of Social Media Use

The 29-item CASM (Multimedia Appendix 1) was produced by the exploratory factor analysis described in study 1.

##### Bergen Facebook Addiction Scale

The 6-item Bergen Facebook Addiction Scale (BFAS) was used to assess self-perceived levels of dependence on the social media platform Facebook [39]. The BFAS is a self-report measure that asks participants to rate their Facebook use over the past week. Items are scored on a 5-point Likert scale from 0 (very rarely) to 5 (very often). The BFAS demonstrates acceptable psychometric properties and has demonstrated validity in additional young adult samples [40,41].

##### Motivations for Electronic Interaction Scale

The Motivations for Electronic Interaction Scale (MEIS) is a 22-item self-report survey measure used to assess an individual’s motivations for using electronic interactions [42]. Items are scored on a 5-point Likert scale from 1 (not at all true) to 5 (extremely true). The Motivations for Electronic Interactions Comparison and Feedback Subscale (MEIS-CF) can be used to assess an individual’s motivation for using electronic interaction based on their desire to make social comparisons. The MEIS-CF was used in this study to assess an individual’s comparison and feedback-seeking behaviors on social media.

##### Brief Fear of Negative Evaluation Scale

The 12-item Brief Fear of Negative Evaluation Scale (B-FNE) was used to measure self-perceived levels of anxiety related to being negatively evaluated by another person [43]. The B-FNE is a self-report measure that asks participants to rate how accurately a statement describes them. Items are scored on a 5-point Likert scale from 1 (not at all characteristic of me) to 5 (extremely characteristic of me). The B-FNE has demonstrated adequate psychometric properties among undergraduate students.

##### Depression, Anxiety, Stress Scale

The Depression, Anxiety, Stress Scale (DASS) is a 21-item self-report measure designed to assess an individual’s emotional state of depression, anxiety, and stress [44]. The DASS asks participants to indicate how much a statement applied to them during the past week. Items are scored on a 4-point Likert scale from 0 (did not apply to me at all) to 3 (applied to me very much or most of the time). The DASS has strong psychometric properties and has consistently demonstrated adequate validity [45].

##### Measure of Verbally Expressed Emotion

The Measure of Verbally Expressed Emotion (MoVEE), a 19-item self-report measure, was used to measure an individual’s comfort in verbally expressing different emotions [46]. The MoVEE asks participants to answer items relating to how comfortable they are expressing feelings to others. Items are scored on a 4-point Likert scale from 0 (strongly disagree) to 4 (strongly agree). The MoVEE demonstrates strong validity and reliability among young adults.

### Procedures

Data collection occurred from December 2018 to December 2019 on the online research platform Qualtrics. After providing informed consent, participants completed a demographic survey and the 29-item CASM. Participants then completed the B-FNES, DASS, MEIS, BFAS, and MoVEE. After completing the study measures, participants received a debriefing form with a list of local and national mental health resources.

### Data Analysis

Descriptive statistics were calculated to characterize sample demographics. Model fit indices calculated included root mean square error of approximation (RMSEA) [47], comparative fit index (CFI) [48], Tucker-Lewis index (TLI) [49,50], and standardized root mean square [51]. RMSEA provides an estimate of how far a hypothesized model is from a perfect model, CFI and TLI compare the performance of a hypothesized model against a null model, and standardized root mean squared residual measures the average difference between observed and predicted correlations.

Discriminant validity was examined by calculating correlations between the CASM, the MoVEE, and self-reported frequency of phone video game use. The MoVEE scale and the frequency of phone video game use question were chosen to assess discriminant validity, as both measure smartphone uses not related to social media. Construct validity was determined by calculating correlations between CASM latent factors, the BFAS, and MEIS-CF. Criterion validity was evaluated by calculating correlations between CASM latent factors and 2 indicators of well-being: fear of negative evaluation (measured by the B-FNE) and depression, anxiety, and stress (measured by the DASS).

Finally, a bivariate correlation was calculated to test whether there is a significant relationship between hours per day spent on social media and symptoms of depression, anxiety, and stress. For all analyses, missing data were handled using listwise deletion. All statistical analyses were conducted with SPSS.

### Ethical Considerations

This study received institutional review board approval from Iona College (reference number: 201718‐22) in 2018. Before participating, individuals agreed to participation by electronically signing a consent form which outlined study aims, procedures, time requirement, potential risks, and rights for human research subjects. Individuals from special or protected groups (ie, individuals who are <18 years old, imprisoned, or cognitively impaired) with impaired ability to provide free consent were ineligible to participate. We did not screen for pregnancy, and pregnant women were considered eligible to participate.

### Results

Table 3 presents the standardized parameter estimates for the confirmatory factor analysis model as well as the factor loading and error variance for each item. A variety of indices of model fit were examined: the chi-squared test of model fit was significant (*χ*²_356_=941, *P*<.001; RMSEA=0.064; CFI=0.855; TLI=0.848; standardized root mean squared residual=0.060). The factors and their internal consistency reliability are as follows: self-branding (0.817), compulsive use (0.771), disruptive use (0.699), impulsive sharing (0.750), social engagement (0.712), induce negative emotions (0.751), and induce positive emotions (0.746). As expected, all CASM factors were positively correlated with one another but not so strongly as to suggest a single factor (Table 4). Skewness and kurtosis of all items and subscales on the CASM are reported in Multimedia Appendix 2.

**Table 3.** Internal consistencies, factor loadings, and error variances for the final 29-item, 7-factor scale from study 2 confirmatory factor analysis (CFA).

Scale (internal consistency) and item	Factor loading	Error variance
Self-branding (0.817)		
How much do your profiles represent an “idealized self”?	0.332	0.110
How often do you put a filter on pictures that you post/share?	0.589	0.347
How important to you is the number of likes you get on a post?	0.711	0.505
To what extent do you post on a schedule/certain time of day, thinking people will be more likely to see your post?	0.710	0.504
To what extent do you have a process/routine to developing your posts before sharing them?	0.810	0.656
How much does the number of likes you get on a post affect your mood?	0.707	0.55
Compulsive use (0.771)		
How often do you check social media during face-to-face conversations?	0.523	0.274
How often do you spend more time on social media than you intended to?	0.710	0.504
How often do you sleep with your phone within arm’s reach?	0.394	0.155
How often do you stop doing other tasks (such as homework) to check social media?	0.732	0.537
How often do you lose track of time because you get so involved in your social media activity?	0.771	0.594
Disruptive use (0.699)		
How hard is it to not check social media during class?	0.566	0.320
How often has a loved one (parent, friends, etc.) told you that you are on social media too often?	0.599	0.359
How often have you gotten “in trouble” in class or at work for using social media when you shouldn’t have been?	0.542	0.294
How likely are you to use (check or post) social media accounts while driving?	0.453	0.206
How uncomfortable do you feel if you are away from your phone and can’t use social media for an extended period of time (ie, one hour or more)?	0.537	0.289
Impulsive sharing (0.750)		
How often have you felt that you “over-shared” on social media?	0.795	0.632
How often have you posted something on a social media site and later regretted it?	0.718	0.515
How often do you post on social media as a reaction to stress?	0.642	0.412
Social engagement (0.712)		
How often do you use social media to stay connected for extracurricular activities?	0.397	0.157
How often do you use social media for information about school/academics?	0.421	0.177
How often do you use social media for activism purposes?	0.788	0.620
How often do you engage in discourse about social issues/justice over social media?	0.771	0.595
Induce negative emotions (0.751)		
Do you ever spend time viewing profiles even though you know it makes you feel badly about yourself?	0.741	0.548
How often do you post to make others think you are feeling better than you actually are?	0.673	0.453
How often do you look at social media pages that you know will make you feel bad/induce negative affect?	0.648	0.420
Induce positive emotions (0.746)		
How often do you turn to social media because you think it will improve your mood?	0.752	0.566
How often do you turn to social media to relax?	0.772	0.596
How much does your social media activity make you feel connected to others?	0.607	0.368

**Table 4.** Correlations between latent Comprehensive Assessment of Social Media Use (CASM) factors, means, and SDs of CASM factors.

	SB^a^	CU^b^	DU^c^	IS^d^	SE^e^	INE^f^	IPE^g^
SB	—^h^	0.42^i^	0.45^i^	0.51^i^	0.36^i^	0.60^i^	0.42^i^
CU	0.42^i^	—	0.55^i^	0.36^i^	0.23^i^	0.39^i^	0.46^i^
DU	0.45^i^	0.55^i^	—	0.42^i^	0.29^i^	0.39^i^	0.37^i^
IS	0.51^i^	0.36^i^	0.42^i^	—	0.31^i^	0.59^j^	0.47^i^
SE	0.36^i^	0.23^i^	0.29^i^	0.31^i^	—	0.33^i^	0.36^i^
INE	0.60^i^	0.39^i^	0.39^i^	0.59^j^	0.33^i^	—	0.44^i^
IPE	0.42^i^	0.46^i^	0.37^i^	0.47^i^	0.36^i^	0.44^i^	—
Total sample, mean (SD)	4.08 (2.09)	5.81 (1.87)	3.31 (1.65)	2.85 (1.95)	3.88 (2.00)	3.44 (2.31)	4.77 (2.25)

a SB: self-branding.

b CU: compulsive use.

c DU: disruptive use.

d IS: impulsive sharing.

e SE: social engagement.

f INE: induce negative emotions.

g IPE: induce positive emotions.

h Not applicable.

i *P*<.01.

j *P*<.05.

The results found all CASM latent factors to be weakly correlated with the MoVEE scores and frequency of phone video game use, providing initial evidence of discriminant validity (Table 5). Scores on the BFAS and MEIS-CF, which both assess social media behavior, were correlated with the CASM, providing initial evidence of construct validity (Table 5). All CASM latent factors were correlated with B-FNE scores. Moreover, all CASM latent factors were found to be correlated with scores on the depression, anxiety, and stress subscales from the DASS, providing initial evidence of criterion validity (Table 5). The internal consistency reliability for measures used in validity testing is as follows: MoVEE (0.385), BFAS (0.802), MEIS-CF (0.924), B-FNE (0.791), and DASS (0.937).

Finally, a bivariate correlation revealed no statistically significant association between hours per day spent on social media and symptoms of depression, anxiety, and stress (*r*_397_=0.06; *P*=.21; *R*^2^=0.003), where less than 1% of the variability in symptoms of depression, anxiety, and stress could be accounted for by hours per day spent on social media.

**Table 5.** Correlations between the Comprehensive Assessment of Social Media Use (CASM) and indicators of validity.

Measure of validity	Self-branding	Compulsive use	Disruptive use	Impulsive sharing	Social engagement	Induce negative emotions	Induce positive emotions
Discriminant							
MoVEE^a^	−0.04	0.02	0.02	−0.10^b^	0.01	−0.16^c^	0.04
Phone video game use	0.07	0.06	0.01	−0.01	−0.18^c^	0.04	−0.05
Convergent							
B-FAS^d^	0.35^c^	0.42^c^	0.41^c^	0.44^c^	0.28^c^	0.45^c^	0.45^c^
MEIS-CF^e^	0.54^c^	0.36^c^	0.37^c^	0.46^c^	0.27^c^	0.60^c^	0.41^c^
Criterion validity							
B-FNE^f^	0.26^c^	0.19^c^	0.15^c^	0.25^c^	0.11^c^	0.40^c^	0.21^c^
DASS^g^—depression	0.10^b^	0.10^b^	0.14^c^	0.25^c^	0.08^b^	0.41^c^	0.10^b^
DASS—anxiety	0.16^c^	0.15^c^	0.20^c^	0.26^c^	0.14^c^	0.39^c^	0.14^c^
DASS—stress	0.25^c^	0.20^c^	0.29^c^	0.34^c^	0.16^c^	0.45^c^	0.19^c^
CASM as an outcome							
Nonsuicidal self-injury	*t*(496)=5.10, *P*<.001^c^	*t*(496)=1.65, *P*=.10	*t*(494)=1.50, *P*=.14	*t*(486)=2.97, *P*<.001^c^	*t*(496)=1.68, *P*=.09	*t*(488)=5.50, *P*<.001^c^	*t*(496)=2.41, *P*=.02^b^

a MoVEE: Measure of Verbally Expressed Emotions.

b *P*<.05.

c *P*<.01.

d B-FAS: Bergen Facebook Addiction Scale.

e MEIS-CF: Motivations for Electronic Interactions Comparison and Feedback Subscale.

f B-FNE: Brief Fear of Negative Evaluation Scale.

g DASS: Depression Anxiety Stress Scale.

## Discussion

### Principal Findings

This study developed and validated the CASM. Exploratory and confirmatory factor analyses resulted in a 29-item self-report survey measure that assesses 7 distinct types of social media behavior: self-branding, compulsive use, disruptive use, impulsive sharing, social behavior, induce negative emotions, and induce positive emotions. Each factor of the CASM demonstrates good internal consistency reliability. Further, following cutoff criteria outlined by Hu and Bentler [52], the CASM demonstrates adequate model fit with moderate discriminant, convergent, and criterion validity.

To meaningfully advance research on social media use and mental health, researchers require better measurement tools to capture a range of online behaviors and engagement patterns. Encouragingly, the CASM measures a range of social media behaviors identified in prior research [53-55] that are clinically meaningful. To summarize, the self-branding subscale evaluates how often an individual engages in self-presentation strategies to manage how others perceive them and is similar to digital status–seeking behavior identified in prior research [54]. Individuals with higher scores on the branding behaviors subscale are more likely to have their own social media “brand,” which they adhere to. The compulsive use subscale measures behavior that suggests an overreliance on social media, which prior research has framed as addictive social media behavior [53]. Individuals with higher scores in this category may find it hard to disconnect from social media. The disruptive use subscale measures how frequently an individual uses social media maladaptively. This factor is distinct from the addictive behavior subscale, as its items assess social media use in inappropriate contexts (eg, during class or while driving), rather than use that is excessive. The impulsive sharing subscale examines how often an individual impulsively posts on social media, which aligns with oversharing behavior characterized in prior research [55]. Individuals with higher scores on the impulsive posting subscale are more likely to “over-share,” or post in an unedited, spontaneous manner that results in feelings of regret. The social engagement subscale examines how often an individual uses social media to communicate with others. Individuals with higher scores in this category are more likely to use social media as a tool to communicate and plan with others. The induce negative emotions subscale explores how often an individual uses social media to worsen their affect. One item in this factor evaluates upward social comparisons, a behavior examined extensively by prior research [56-58]. Individuals with higher scores in this category are more likely to use social media to lower their mood. Finally, the induce positive emotions subscale examines how often an individual uses social media to improve their affect. Individuals with higher scores in this category are more likely to use social media as a coping strategy to feel more relaxed or connected with others.

The CASM is novel in that it is one of the first validated measures to assess both positive and negative aspects of social media use simultaneously. Social media use measures have traditionally focused on overtly negative patterns of online engagement [27,28,32,41], such as addictive characteristics. The CASM extends this research by also measuring overtly positive aspects of social media use, such as social connection. This inclusion is especially relevant to advancing our understanding of social media and mental health; emerging research shows that social media may promote mental health through increased social support and peer connection [59]. However, existing evidence is still limited, emphasizing the need for improved measurement to assess potentially protective aspects of social media use.

Another advantage of the CASM is its emphasis on reporter agreement with items, rather than duration or frequency reports of social media engagement. When examining correlations between the CASM and indicators of mental well-being, self-perceived levels of depression, anxiety, and stress were significantly associated with the induce negative emotion and impulsive sharing subscales (Table 5). Notably, self-perceived levels of depression, anxiety, and stress as measured by the DASS were not associated with the self-reported hours per day spent on social media. This finding aligns with prior research showing that characterizing social media use as specific behaviors, rather than time spent online, is a better predictor of mental health [5,18,19].

Advancing our understanding of social media use is especially important for clinicians and parents tasked with promoting the well-being of young people. It is our hope that researchers will leverage the CASM’s ability to measure distinct aspects of social media behavior simultaneously to identify unique “engagement profiles” that more accurately reflect how people interact with social media. With a more holistic understanding of how an individual uses social media, it may be possible to gain deeper insight into what specific patterns of use may be adaptive, neutral, or detrimental to mental health. With this information, interventions and guidance aimed at promoting online hygiene could become more targeted and personalized. To accomplish this, future research should continue to advance the measurement of social media use, incorporating additional markers, such as passive sensing, to more accurately capture youth engagement with these platforms.

### Limitations

The findings from this initial validation study are important and yield ideas for further research. This study used a convenience sample of college students, limiting the generalizability of the findings beyond this population. This is especially important given demonstrable age differences in social media use [60]. Future research should examine the CASM’s application and validity across different age groups. Second, the CASM is a self-report measure and is therefore susceptible to response bias. This may have resulted in skewed responses, especially on items that conveyed negative social media habits. Future research should seek novel ways to assess social media use without the use of self-report measures. Third, while the 7 categories of social media use identified in this study cover a broad range of behaviors, it is likely that there are other clinically relevant dimensions of social media use. Future research should continue to examine and identify novel ways individuals interact and use social media, especially as these platforms continue to extend into news, gaming, and entertainment ecosystems and incorporate advances in artificial intelligence. Finally, this study predates the COVID-19 pandemic. Given documented increases in social media use during the pandemic [61], the behaviors identified in this study may not reflect social media engagement in more recent populations.

### Conclusions

This study led to the development of the CASM, a 29-item scale that assesses 7 distinct social media behaviors: self-branding, compulsive use, disruptive use, impulsive sharing, social engagement, induce negative emotions, and induce positive emotions. The CASM demonstrates adequate model fit with moderate discriminant, convergent, and criterion validity. The CASM is one of the first validated measures to multiple, distinct aspects of social media behavior simultaneously, enabling researchers to more effectively examine associations between social media use and mental health outcomes. With this improved measurement, researchers can better assess how individuals use social media, and how this use may impact mental well-being.

## Supplementary material

10.2196/87599 Multimedia Appendix 1 Comprehensive Assessment of Social Media Use (CASM).

10.2196/87599 Multimedia Appendix 2 Summary of skewness and kurtosis.

## Abbreviations

B-FNE: Brief Fear of Negative Evaluation Scale
BFAS: Bergen Facebook Addiction Scale
CASM: Comprehensive Assessment of Social Media Use
CFI: comparative fit index
DASS: Depression, Anxiety, Stress Scale
LGBTQ: lesbian, gay, bisexual, transgender/transsexual, and queer
MEIS: Motivations for Electronic Interaction Scale
MEIS-CF: Motivations for Electronic Interactions Comparison and Feedback Subscale
MoVEE: Measure of Verbally Expressed Emotion
RMSEA: root mean square error of approximation
TLI: Tucker-Lewis index
