# Supplementary material for: The Comprehensive Assessment of Social Media Use: Development and Validation Study
Source: JMIR Form Res. 2026 Apr 29;10:e87599. doi: 10.2196/87599 (PMC13128055; doi:10.2196/87599)
Supplement: Multimedia Appendix 1 [file formative-v10-e87599-s001.pdf]

Comprehensive Assessment of Social Media Use (CASM)

Directions: Please use the scale below to answer the following questions. Use either the frequency anchors or the intensity anchors, whichever applies best to each question.

1	2	3	4	5	6	7	8	9	10
Not at all/Not Often			Somewhat/Sometimes				Very Much/Very Often		

1. How much do your profiles represent an "idealized self"?
2. How often do you put a filter on pictures that you post/share?
3. How important to you is the number of likes you get on a post?
4. To what extent do you post on a schedule/certain time of day, thinking people will be more likely to see your post?
5. To what extent do you have a process/routine to developing your posts before sharing them?
6. How much does the number of likes you get on a post affect your mood?
7. How often do you check social media during face-to-face conversations?
8. How often do you spend more time on social media that you intended to?
9. How often do you sleep with your phone within arm's reach?
10. How often do you stop doing other tasks (such as homework) to check social media?
11. How often do you lose track of time because you get so involved in your social media activity?
12. How hard is it to not check social media during class?
13. How often has a loved one (parent, friends, etc.) told you that you are on social media too often?
14. How often have you gotten in trouble in class or at work for using social media when you shouldn't have been?
15. How likely are you to use (check or post) social media accounts while driving?
16. How uncomfortable do you feel if you are away from your phone and can't use social media for an extended period of time (i.e. one hour or more)?
17. How often have you felt that you "over-shared" on social media?
18. How often have you posted something on social media site and later regretted it?
19. How often do you post on social media as reaction to stress?
20. How often do you use social media to stay connected for extracurricular activities?
21. How often do you use social media for information about school/academics?
22. How often do you use social media for activism purposes?
23. How often to you engage in discourse about social issues/justice over social media?
24. Do you ever spend time viewing profiles even though you know it makes you feel badly about yourself?
25. How often do you post to make others think you are feeling better than you actually are?
26. How often do you look at social media pages that you know will make you feel bad/induce negative affect?
27. How often do you turn to social media because you think it will improve your mood?
28. How often do you turn to social media to relax?
29. How much does your social media activity make you feel connected to others?

Scoring Instructions: Items for each subscale should be averaged. There are no reverse scored items.

Self-Branding (6 items): 1, 2, 3, 4, 5, 6
Compulsive Use (5 items): 7, 8, 9, 10, 11
Disruptive Use (5 items): 12, 13, 14, 15, 16
Impulsive Sharing (3 items): 17, 18, 19
Social Engagement (4 items): 20, 21, 22, 23
Induce Negative Emotions (3 items): 24, 25, 26
Induce Positive Emotions (3 items): 27, 28, 29
