# Supplementary material for: The Comprehensive Assessment of Social Media Use: Development and Validation Study
Source: JMIR Form Res. 2026 Apr 29;10:e87599. doi: 10.2196/87599 (PMC13128055; doi:10.2196/87599)
Supplement: Multimedia Appendix 2 [file formative-v10-e87599-s002.docx]

| **Supplemental Table 1**: Summary of Skewness and Kurtosis | | | | |
| --- | --- | --- | --- | --- |
| CASM Item / Subscale | Skewness | | Kurtosis | |
|  | Statistic | Std. Error | Statistic | Std. Error |
| Item 1 | .248 | .109 | -1.158 | .218 |
| Item 2 | .377 | .110 | -1.225 | .219 |
| Item 3 | .480 | .109 | -.771 | .218 |
| Item 4 | .470 | .110 | -1.258 | .219 |
| Item 5 | .910 | .110 | -.403 | .219 |
| Item 6 | 1.093 | .110 | .193 | .220 |
| Item 7 | 1.027 | .110 | .837 | .219 |
| Item 8 | .093 | .109 | -.851 | .217 |
| Item 9 | -1.418 | .108 | .664 | .217 |
| Item 10 | -.232 | .109 | -.909 | .217 |
| Item 11 | .109 | .108 | -1.052 | .217 |
| Item 12 | .497 | .110 | -.820 | .219 |
| Item 13 | .833 | .111 | -.324 | .222 |
| Item 14 | 1.737 | .113 | 2.845 | .225 |
| Item 15 | 2.077 | .113 | 3.810 | .226 |
| Item 16 | .818 | .110 | -.281 | .220 |
| Item 17 | 1.591 | .111 | 1.999 | .222 |
| Item 18 | 1.136 | .110 | .591 | .220 |
| Item 19 | 1.394 | .111 | .924 | .222 |
| Item 20 | .313 | .109 | -1.078 | .218 |
| Item 21 | .401 | .109 | -.892 | .218 |
| Item 22 | 1.071 | .111 | .178 | .222 |
| Item 23 | 1.136 | .111 | .290 | .221 |
| Item 24 | .660 | .110 | -.884 | .220 |
| Item 25 | .907 | .111 | -.440 | .221 |
| Item 26 | 1.338 | .111 | .833 | .222 |
| Item 27 | .500 | .109 | -.853 | .218 |
| Item 28 | .121 | .108 | -.956 | .216 |
| Item 29 | .250 | .109 | -.810 | .217 |
|  |  |  |  |  |
| Self-Branding | .451 | .108 | -.642 | .216 |
| Compulsive Use | -.193 | .108 | -.244 | .216 |
| Disruptive Use | .701 | .109 | .063 | .217 |
| Impulsive Sharing | 1.250 | .110 | 1.255 | .219 |
| Social Engagement | .548 | .108 | -.135 | .217 |
| Induce Negative Emotions | .765 | .110 | -.322 | .219 |
| Induce Positive Emotions | .334 | .108 | -.494 | .216 |
